# Waking up to the truth: Associations between sleep disorders and multidomain functional outcomes in Alzheimer's disease

**DOI:** 10.1002/alz.71200

**Published:** 2026-02-24

**Authors:** Chao Tang, Jiaxin Yan, Yi Chen, Xiaoxue Peng, Xiaoyang Lei, Ming Zhang, Dian He

**Affiliations:** ^1^ Department of Neurology Affiliated Hospital of Guizhou Medical University Guiyang Guizhou Province China

**Keywords:** Alzheimer's disease, cognitive impairment, multidomain functional impairments, sleep disorders, structural equation modeling

## Abstract

**INTRODUCTION:**

Sleep disorders are common in older adults and have been increasingly linked to cognitive dysfunction in Alzheimer's disease (AD); however, their associations with functional outcomes and underlying pathways remain insufficiently characterized.

**METHODS:**

We performed a cross‐sectional analysis of 10,823 participants from the National Alzheimer's Coordinating Center database, examining associations between sleep disorders and instrumental activities of daily living (IADL) using regression, structural equation modeling (SEM), and mediation analyses.

**RESULTS:**

Sleep disorders were significantly associated with greater cognitive impairment and poorer IADL performance among individuals with AD. Mediation analysis indicated that cognitive impairment accounted for ≈62% of the association between sleep disorders and IADL difficulties, suggesting a substantial indirect association through cognitive function.

**DISCUSSION:**

Sleep disorders are strongly associated with cognitive impairment and functional limitations in AD. These findings highlight the potential clinical value of screening and managing sleep problems as part of comprehensive care strategies.

## Highlights

- Sleep disorders significantly associated with cognitive impairment in patients with Alzheimer's disease.
- Cognitive impairment mediates the relationship between sleep disorders and functional decline.
- Sleep apnea shows strongest association with cognitive decline in AD.
- Rapid eye movement (REM) sleep behavior disorder significantly correlates with instrumental activities of daily living (IADL) impairment.
- Treating sleep disorders may preserve cognitive function and daily living abilities.

## 1 BACKGROUND

Alzheimer's disease (AD) represents a significant global health crisis affecting millions, characterized by a progressive decline in cognitive functions such as memory and reasoning. As the leading cause of dementia, AD poses substantial challenges not only to individuals diagnosed with the disease but also to caregivers and health care systems.^1^ The complexity of AD's etiology necessitates a comprehensive approach to understanding its multifactorial nature, with emerging research highlighting the critical role of sleep disorders in exacerbating cognitive decline.^2^, ^3^

Adequate sleep is vital for maintaining cognitive health, yet individuals with AD frequently experience sleep disorders, including insomnia and sleep apnea.^2^, ^4^ These disorders compromise sleep quality and may accelerate cognitive impairment through mechanisms such as impaired memory consolidation and heightened inflammation.^5^, ^6^ Notably, the interaction between poor sleep and cognitive dysfunction can create a cyclical pattern where cognitive deficits further disrupt sleep, leading to an overall decline in health and well‐being.^7^

Research indicates that sleep disorders have significant implications for daily functioning, particularly in performing instrumental activities of daily living (IADL).^8^ Cognitive impairments often impair the ability of individuals to manage daily tasks, such as financial management, meal preparation, and social engagement.^9^ This raises concerns about independence and quality of life in patients with AD, as functional decline compounds the psychological and social impacts of cognitive deterioration.^10^

Despite the documented links between sleep quality, cognitive function, and daily activities, a nuanced understanding of these relationships remains crucial. Recent studies have utilized various analytical techniques to explore how sleep disorders influence cognitive and functional outcomes in AD.^11^ For instance, mediation analyses have illustrated that cognitive impairment may be associated with the relationship between sleep issues and IADL deficits, indicating that enhancing sleep quality could be a viable avenue for improving cognitive health and functional capabilities.^12^, ^13^

Recognizing the interplay between sleep disorders and cognitive health is essential for developing effective intervention strategies.^14^ The existing literature indicates that sleep disturbances are prevalent among individuals with AD, adversely affecting cognitive performance and daily functioning.^15^ For instance, studies suggest that sleep disorders contribute to the acceleration of cognitive decline, highlighting a critical knowledge gap in understanding these complex interactions.^16^

Despite the growing recognition of the link between sleep and cognitive health, much remains to be explored regarding the underlying mechanisms through which sleep disorders exacerbate cognitive impairment and functional deficits in AD.^17^ Although improving sleep may alleviate direct symptoms, it could also enhance cognitive performance, thereby supporting better functional outcomes. This necessitates a focused investigation into how sleep disorders influence cognitive and functional health in individuals affected by AD.^18^, ^19^

This study aims to clarify the intricate relationships between sleep disorders, cognitive function, and functional impairments in individuals with AD. We hypothesize that sleep disorders significantly impact cognitive performance and daily activities, with cognitive impairment acting as a mediating factor. By employing advanced statistical methodologies, including structural equation modeling (SEM) and mediation analysis, we will elucidate the pathways through which sleep disorders influence cognitive health. Ultimately this research seeks to contribute valuable insights into clinical practices and therapeutic strategies, thereby enhancing the quality of life for individuals with AD.

## 2 METHODS

### 2.1 Study design and data source

This cross‐sectional study utilized data from the National Alzheimer's Coordinating Center (NACC) Uniform Data Set (UDS), a standardized clinical assessment protocol implemented across 39 Alzheimer's Disease Research Centers (ADRCs) throughout the United States. The NACC UDS represents a comprehensive longitudinal database systematically collecting demographic, clinical, cognitive, and functional assessments from participants since 2005.^20^ This analysis employed UDS Version 3.0 data collected from September 2015 onward, which includes enhanced assessments for sleep disorders and refined evaluations of functional capacities.

The UDS database includes both cognitively normal individuals and those exhibiting various degrees of cognitive impairment, providing a robust platform for investigating relationships between sleep disorders and functional outcomes across the cognitive spectrum. Data collection follows standardized protocols with rigorous quality control measures, including the centralized training of research personnel, annual certification requirements, and systematic data verification procedures to ensure consistency across participating centers.

**RESEARCH IN CONTEXT**

1. **Systematic review**: We conducted a comprehensive literature review examining the relationships between sleep disorders, cognitive impairment, and functional decline in Alzheimer's disease (AD). Our analysis of existing evidence revealed significant associations between sleep disorders (particularly sleep apnea and rapid eye movement [REM] sleep behavior disorder) and cognitive decline, as well as impairments in instrumental activities of daily living (IADL). However, critical gaps remain in understanding the complex pathways through which sleep disorders influence both cognitive and functional outcomes, particularly the mediating mechanisms that connect these domains.
2. **Interpretation**: Our findings demonstrate that sleep disorders, especially sleep apnea (β = 0.638, *p* < 0.001) and REM sleep behavior (β = 0.289, *p* = 0.004), are significantly associated with cognitive impairment and functional decline in patients with AD. Mediation analysis reveals that cognitive impairment serves as a key mediator, accounting for substantial portions of the relationship between sleep disorders and IADL deficits. These results emphasize that addressing sleep disorders may offer a viable therapeutic avenue for preserving cognitive function and enhancing daily living capabilities in patients with AD.
3. **Future directions**: Several critical research questions emerge from our study. Longitudinal investigations are needed to establish the temporal relationships and directionality between sleep quality changes and AD progression. Further research should explore the underlying biological mechanisms, including neuroinflammation, oxidative stress, and vascular pathways linking sleep disorders to neurodegeneration. In addition, intervention studies examining whether treating sleep disorders can slow cognitive decline and improve functional outcomes are essential for translating these findings into clinical practice.

### 2.2 Study population and selection criteria

The study population comprised adults 50 years of age and older who completed comprehensive UDS evaluations at participating ADRCs.

Inclusion criteria were strictly applied to ensure data completeness and analytical validity:

1. completion of initial UDS visit with complete demographic data captured through Form A1, including age, sex, race, ethnicity, education level, and living situation;
2. availability of sleep disorder assessment data from Form A5 (subject health history) and Form D2 (clinician judgment), encompassing detailed sleep history, sleep disorder diagnoses, and sleep medication usage;
3. completion of cognitive assessment data from Forms C1 and C2, ensuring a thorough evaluation of cognitive health across multiple domains; and
4. functional assessment completion through Form B7 (Functional Activities Questionnaire), capturing IADL [instrumental activities of daily living] performance across 10 standardized domains.

Exclusion criteria were designed to optimize data quality and analytical interpretability:

1. participants with incomplete sleep disorder assessment data, defined as missing responses to more than 50% of sleep‐related items across relevant forms;
2. individuals missing primary outcome variables of interest, including cognitive testing or functional assessments;
3. participants with diagnosed conditions affecting reliable assessment, such as severe aphasia or advanced dementia (CDR Sum of Boxes greater than 2) that would interfere with evaluation validity; and
4. individuals with significant missing data across multiple assessment domains, operationally defined as missing data in three or more major categories (demographic, sleep, cognitive, motor, or functional domains).

(Details of all participants can be found in Table S1.)

### 2.3 Assessment of variables and measurements

Sleep disorders were systematically defined using standardized UDS criteria implemented across all participating centers. Rapid eye movement sleep behavior was assessed through structured clinical interviews focusing on dream enactment behaviors, validated questionnaires administered to both participants and informants, and review of any available polysomnographic findings. Sleep apnea determination included clinical evaluations regarding snoring patterns, witnessed apnea events, and daytime somnolence, supplemented where available by documented sleep studies.

Cognitive function assessment entailed multiple validated instruments, including the Montreal Cognitive Assessment (MoCA),^21^ the Global Clinical Dementia Rating (or CDR Sum of Boxes),^22^ and Trail Making Tests (TMTs).^23^ These evaluations captured various cognitive domains such as executive function, attention, and memory. Multidomain functional assessments focused on IADL were systematically examined using the Functional Activities Questionnaire (FAQ),^24^ which investigated capability in financial management, shopping, meal preparation, medication management, and social engagement.

### 2.4 Missing data management

To address missing data, we employed Multiple Imputation with Chained Equations (MICE), implemented through the mice package in R. This approach included all predictor variables, outcome measures, and auxiliary variables with moderate correlations (greater than 0.4) to the missing data, generating five imputed datasets. The results were pooled using Rubin's rules, allowing for valid statistical inferences.^25^

To assess the robustness of our findings, we conducted sensitivity analyses comparing complete case analysis—where incomplete cases were excluded—with results from the imputed datasets. This comparison allows us to evaluate potential biases introduced by the missing data and the impact of different handling methods on study outcomes. While MICE helps mitigate bias under the assumption that data are missing at random (MAR), sensitivity analyses ensure transparency and integrity in data processing, ultimately contributing to reliable conclusions regarding the relationships among sleep disorders, cognitive impairment, and functional outcomes in AD.

### 2.5 Statistical analysis

Statistical analyses were conducted using R software version 4.3.0. The dataset was composed of participants diagnosed with AD and a control group, focusing on the demographic and clinical characteristics of these individuals. Descriptive statistics for participant characteristics were summarized, including demographic information, clinical features, and performance measures across cognitive and functional domains. Continuous variables were reported as means and standard deviations (SDs) for normally distributed data, whereas non‐normally distributed data were reported using medians and interquartile ranges (IQRs). Categorical variables were presented as frequencies and percentages.

### 2.6 Comparing groups

Between‐group comparisons were made using independent *t*‐tests for continuous variables that met normality assumptions and the Mann–Whitney *U* test for those that did not. Categorical variables were assessed using chi‐square tests or Fisher's exact tests, as appropriate. Effect sizes were estimated to provide context on the practical significance of the findings, employing Cohen's *d* for continuous measures and Cramér's V for categorical measures to assess the strength of associations.

### 2.7 Logistic regression analysis

Logistic regression was used to analyze the association between the presence of sleep disorders and binary outcomes related to cognitive impairment. The model incorporated covariates such as age, gender, and education level to control for potential confounders. This analysis provided insights into how sleep disorders correlate with cognitive health status in the context of AD.

### 2.8 Ordinal regression analysis

In addition to logistic regression, ordinal regression analysis was employed to investigate the relationship between sleep disorders and the severity of cognitive impairment. The CDR Sum of Boxes served as the ordinal outcome variable, allowing the assessment of the likelihood of higher degrees of cognitive impairment based on sleep disorder diagnoses.

### 2.9 Multivariate and machine learning analysis

For predictive insights, a range of multivariate machine learning techniques, including Random Forest and Support Vector Machines (SVMs), were employed to predict AD status based on cognitive performance, functional characteristics, sleep disorders, and relevant demographic and clinical variables. These models are well suited for capturing complex nonlinear relationships and interactions among predictors that may not be adequately represented in traditional regression analyses.

Model performance was evaluated using multiple complementary metrics, including accuracy, precision, recall, F1 score, and the area under the receiver‐operating characteristic (ROC) curve (AUC). To reduce the risk of overfitting and enhance model generalizability, k‐fold cross‐validation was applied. Specifically, the dataset was randomly partitioned into k subsets, with models trained on k‐1 folds and tested on the remaining fold. This process was repeated until each subset served as the validation set.

In addition, feature importance analysis was conducted using the Random Forest model to quantify the relative contribution of each predictor to AD classification. Variable importance was estimated based on the mean decrease in impurity, enabling the identification of factors that most strongly contributed to the discrimination between individuals with and without AD. These analyses were intended to be predictive rather than causal and were used to complement regression‐based findings.

### 2.10 Structural equation modeling

SEM was employed to examine the associations among sleep disorders, cognitive impairment, and multidomain functional outcomes within a unified analytical framework. SEM enables the simultaneous estimation of multiple relationships while accounting for measurement error in latent or composite psychological and functional constructs.

In the model, cognitive and functional status was characterized using the CDR score, CDR total score, and multiple indicators of IADL. Path coefficients were reported as standardized regression coefficients (β), and all associations were interpreted in relation to the predefined variable coding schemes.

Model fit was evaluated using multiple complementary indices, including the Comparative Fit Index (CFI), with values greater than 0.95 indicating good model fit; the Root Mean Square Error of Approximation (RMSEA), with values below 0.06 reflecting adequate fit; and the Standardized Root Mean Square Residual (SRMR), where values less than 0.08 were considered acceptable.

To reduce potential confounding, the SEM incorporated relevant covariates, including the use of anti‐AD medications, sleep aids, and antipsychotic drugs, as well as comorbid conditions such as cardiovascular disease, diabetes, and obesity. All estimated pathways reflect statistical associations rather than causal effects, consistent with the cross‐sectional design of the study.^26^

### 2.11 Chain mediation analysis

Chain mediation analysis was conducted to examine the indirect associations linking sleep disorders, cognitive impairment, and functional outcomes within a single analytical framework. This approach was used to assess whether cognitive impairment statistically mediates the association between sleep disorders and functional limitations, as measured by IADL.

The mediation model was specified to estimate both the direct association between sleep disorders and functional outcomes and the indirect associations operating through cognitive impairment. Indirect effects were quantified using standardized coefficients and interpreted in accordance with the predefined variable coding schemes.

To obtain robust confidence intervals for the indirect associations, nonparametric bootstrapping procedures were applied. This resampling approach accounts for the typically non‐normal distribution of indirect effects and enhances the stability and reliability of the estimated mediation paths.

Relevant covariates, including medication use and comorbid conditions, were incorporated into the mediation model to reduce potential confounding. All mediation results were interpreted as statistical associations rather than causal effects, consistent with the cross‐sectional design of the study.^27^

### 2.12 Directed acyclic graph methodology

Directed acyclic graph (DAG) analysis played a crucial role in mapping out the hypothesized causal relationships among sleep disorders, cognitive impairments, and functional outcomes.^28^ The DAG was constructed to represent the assumed relationships, providing guidance for the analytical approach. This visualization clarified the direct and indirect pathways under investigation, supporting the design of both the SEM and mediation analyses.

### 2.13 Ethical considerations

This study utilized de‐identified data sourced from the NACC database. Written informed consent was obtained from participants at each ADRC, ensuring that all individuals were fully aware of their involvement in the research. Ethical approval was granted by the institutional review boards (IRBs) of each participating ADRC, adhering to the highest ethical standards.

Although the analysis involved a de‐identified dataset, which minimizes risks to individual privacy, IRB approval was considered unnecessary according to federal regulations regarding the use of secondary data. Data access was conducted under strict data use agreements that outline the responsibilities and limitations related to data handling. Comprehensive privacy protections and confidentiality were maintained through established anonymization protocols within the NACC, ensuring the ethical integrity of the study and safeguarding participants’ rights.

## 3 RESULTS

### 3.1 Demographic and clinical characteristics of participants

In this study involving a total of 10,823 participants, the mean age was 69.99 years (SD = 8.93), with a notable difference between groups; those diagnosed with AD had a higher mean age of 71.60 years (SD = 8.92) compared to 68.75 years (SD = 8.73) in the control group, a difference that was statistically significant (*t* = −16.6312, *p* < 0.001) (Table 1). Gender distribution also revealed a significant disparity, with a higher percentage of male participants in the AD group (48.44%) compared to the control group (35.51%), as indicated by a chi‐square test result (χ^2^ = 183.7561, *p* < 0.001). Educational attainment was similar in both groups, with a median of 16 years of education, whereas tobacco use did not present significant differences (χ^2^ = 0.0531, *p* = 0.818). However, alcohol consumption was slightly higher in controls compared to those with AD, with 3.70% versus 4.53% reporting usage, respectively (χ^2^ = 4.6931, *p* = 0.03).

**TABLE 1:** Demographic and clinical characteristics of patients with and without Alzheimer's disease.

Variable	Total (*n *= 10,823)	Control	Alzheimer diagnosis	Statistics	*p*‐value
Age, mean ± SD	69.99 ± 8.93	68.75 ± 8.73	71.60 ± 8.92	*t *= −16.631^2^	<0.001
Gender, *n* (%)				χ^2 ^= 183.756^1^	<0.001
Male	4454 (41.15)	2167 (35.51)	2287 (48.44)		
Female	6369 (58.85)	3935 (64.49)	2434 (51.56)		
Education, median (Q_1_, Q_3_)	16.00 (14.00, 18.00)	16.00 (15.00, 18.00)	16.00 (14.00, 18.00)	*Z =* 12.258^3^	<0.001
Tobacco use, *n* (%)				χ^2 ^= 0.053^1^	0.818
None	6783 (62.67)	3830 (62.77)	2953 (62.55)		
Yes	4040 (37.33)	2272 (37.23)	1768 (37.45)		
Alcohol use, *n* (%)				χ^2 ^= 4.693^1^	0.03
None	10,383 (95.93)	5876 (96.30)	4507 (95.47)		
Yes	440 (4.07)	226 (3.70)	214 (4.53)		
*APOE* status, *n* (%)				*Z =* −16.417^3^	<0.001
ε3, ε3	5216 (48.19)	3414 (55.95)	1802 (38.17)		
ε3, ε4	3556 (32.86)	1615 (26.47)	1941 (41.11)		
ε3, ε2	898 (8.30)	638 (10.46)	260 (5.51)		
ε4, ε4	852 (7.87)	255 (4.18)	597 (12.65)		
ε4, ε2	258 (2.38)	145 (2.38)	113 (2.39)		
ε2, ε2	43 (0.40)	35 (0.57)	8 (0.17)		
*APOE* ε4 alleles, *n* (%)				*Z =* −25.390^3^	<0.001
No ε4 allele	6157 (56.89)	4087 (66.98)	2070 (43.85)		
1 copy of ε4 allele	3814 (35.24)	1760 (28.84)	2054 (43.51)		
2 copies of ε4 allele	852 (7.87)	255 (4.18)	597 (12.65)		
Sleep apnea, *n* (%)				χ^2 ^= 35.484^1^	<0.001
None	10,512 (97.13)	5978 (97.97)	4534 (96.04)		
Yes	311 (2.87)	124 (2.03)	187 (3.96)		
REM sleep behavior, *n* (%)				χ^2 ^= 6.783^1^	0.009
None	10,459 (96.64)	5921 (97.03)	4538 (96.12)		
Yes	364 (3.36)	181 (2.97)	183 (3.88)		
Other sleep issues, *n* (%)				χ^2 ^= 17.922^1^	<0.001
None	7550 (69.759)	4357 (71.403)	3193 (67.634)		
Yes	3273 (30.241)	1745 (28.597)	1528 (32.366)		
Death, *n* (%)				χ^2 ^= 711.529^1^	<0.001
None	9803 (90.58)	5929 (97.16)	3874 (82.06)		
Yes	1020 (9.42)	173 (2.84)	847 (17.94)		

*Note*: This table summarizes the demographic and clinical characteristics of the study population, including age, gender, education, tobacco and alcohol use, *APOE* ε4 status, sleep apnea, REM sleep behavior, other sleep issues, and mortality status, comparing individuals diagnosed with Alzheimer's disease to those in the control group.

Abbreviations: *APOE*, apolipoprotein E; REM, rapid eye movement.

The *APOE* ε4 allele status showed significant differences between groups, with 41.11% of patients with Alzheimer's carrying the allele compared to 26.47% in controls (*Z =* −16.4173, *p* < 0.001). In addition, a greater proportion of the total cohort (56.89%) possessed two copies of the ε4 allele, with a notable difference between groups observed in the frequency of individuals carrying these alleles. Sleep disorders were also assessed, revealing that 2.87% of participants experienced sleep apnea, with significantly higher rates of 3.96% reported in the AD group as opposed to 2.03% in controls (χ^2^ = 35.4841, *p* < 0.001). Although REM sleep behavior was identified in 3.36% of participants, this did not display significant group differences (χ^2^ = 6.7831, *p* = 0.009). Other sleep issues were prevalent in 30.24% of participants, and 32.37% of individuals in the AD group reported such issues, contrasting with 28.60% in the control group (χ^2^ = 17.9221, *p* < 0.001). Finally, mortality status reflected pronounced differences, with 90.58% of the total cohort reporting no deaths; yet this rate fell to 82.06% in the AD group, indicating the substantial impacts of AD on patient survival (χ^2^ = 711.5291, *p* < 0.001).

### 3.2 Cognitive and psychiatric assessments in patients with AD

Among 10,823 participants, significant differences in cognitive and psychiatric measures were observed between individuals with AD and controls (Table 2). Controls had a median CDR Total score of 0.00 (Q1 = 0.00, Q3 = 0.00), whereas AD patients showed a higher median of 0.50 (Q1 = 0.50, Q3 = 1.00; *Z =* −85.48, *p* < 0.001). Consistently, the CDR Total Score demonstrated marked group differences, with a median of 0.00 (Q1 = 0.00, Q3 = 0.00) in controls and 3.00 (Q1 = 1.00, Q3 = 5.00) in the AD group (*Z =* −87.20, *p* < 0.001).

**TABLE 2:** Cognitive scores and psychiatric evaluations in Alzheimer's disease patients compared to controls.

Variable	Total (*n *= 10,823)	Control	Alzheimer diagnosis	Statistics	*p*‐value
Global CDR score, M (Q1, Q3)	0.00 (0.00, 0.50)	0.00 (0.00, 0.00)	0.50 (0.50, 1.00)	*Z =* −85.483^3^	<0.001
CDR Sum of Boxes, median (Q1, Q3)	0.50 (0.00, 2.50)	0.00 (0.00, 0.00)	3.00 (1.00, 5.00)	*Z =* −87.196^3^	<0.001
Animal Naming Total, median (Q1, Q3)	18.00 (13.00, 23.00)	21.00 (18.00, 25.00)	13.00 (9.00, 18.00)	*Z =* 60.527^3^	<0.001
Vegetable Naming Total, M (Q1, Q3)	12.00 (8.00, 16.00)	15.00 (12.00, 17.00)	8.00 (5.00, 11.00)	*Z =* 62.809^3^	<0.001
TMT‐A time, median (Q1, Q3)	34.00 (26.00, 48.00)	29.00 (23.00, 37.00)	45.00 (32.00, 72.00)	*Z =* −48.703^3^	<0.001
TMT‐A errors, mean ± SD	23.48 ± 2.87	23.99 ± 0.21	22.82 ± 4.24	*t *= 19.032^2^	<0.001
TMT‐A correct, median (Q1, Q3)	90.00 (65.00, 155.00)	72.00 (56.00, 95.00)	156.00 (95.00, 300.00)	*Z =* −59.834^3^	<0.001
TMT‐B time, median (Q1, Q3)	0.00 (0.00, 2.00)	0.00 (0.00, 1.00)	1.00 (0.00, 3.00)	*Z =* −36.927^3^	<0.001
TMT‐B errors, median (Q1, Q3)	24.00 (24.00, 24.00)	24.00 (24.00, 24.00)	24.00 (17.00, 24.00)	*Z =* 46.014^3^	<0.001
TMT‐B correct, median (Q1, Q3)	25.00 (20.00, 27.00)	27.00 (25.00, 28.00)	20.00 (15.00, 23.00)	*Z =* 64.614^3^	<0.001
MoCA Total score, mean ± SD	14.63 ± 3.03	15.54 ± 1.31	13.45 ± 4.05	*t *= 34.011^2^	<0.001
UDS Benson copy Total, median (Q1, Q3)	9.00 (5.00, 12.00)	12.00 (10.00, 13.75)	4.00 (0.00, 8.00)	*Z =* 70.392^3^	<0.001
UDS Benson delay Total, *n* (%)				χ^2 ^= 1385.967^1^	<0.001
None	2284 (21.10)	504 (8.26)	1780 (37.70)		
Yes	8539 (78.90)	5598 (91.74)	2941 (62.30)		
Quality of life satisfaction, *n* (%)				χ^2 ^= 92.637^1^	<0.001
None	9793 (90.48)	5667 (92.87)	4126 (87.40)		
Yes	1030 (9.52)	435 (7.13)	595 (12.60)		
Activity dropout, *n* (%)				χ^2 ^= 272.980^1^	<0.001
None	9032 (83.45)	5409 (88.64)	3623 (76.74)		
Yes	1791 (16.55)	693 (11.36)	1098 (23.26)		
Empty needs, *n* (%)				χ^2 ^= 85.613^1^	<0.001
None	10,221 (94.44)	5872 (96.23)	4349 (92.12)		
Yes	602 (5.56)	230 (3.77)	372 (7.88)		
Boredom, *n* (%)				χ^2 ^= 149.021^1^	<0.001
None	9457 (87.38)	5541 (90.81)	3916 (82.95)		
Yes	1366 (12.62)	561 (9.19)	805 (17.05)		
Mood spirits, *n* (%)				χ^2 ^= 25.448^1^	<0.001
None	10,180 (94.06)	5801 (95.07)	4379 (92.76)		
Yes	643 (5.94)	301 (4.93)	342 (7.24)		
Fearfulness, *n* (%)				χ^2 ^= 64.738^1^	<0.001
None	9637 (89.04)	5563 (91.17)	4074 (86.30)		
Yes	1186 (10.96)	539 (8.83)	647 (13.70)		
Happiness, *n* (%)				χ^2 ^= 17.651^1^	<0.001
None	9838 (90.90)	5609 (91.92)	4229 (89.58)		
Yes	985 (9.10)	493 (8.08)	492 (10.42)		
Helplessness, *n* (%)				χ^2 ^= 183.818^1^	<0.001
None	10,010 (92.49)	5828 (95.51)	4182 (88.58)		
Yes	813 (7.51)	274 (4.49)	539 (11.42)		
Preference to stay home, *n* (%)				χ^2 ^= 0.463^1^	0.496
None	8205 (75.81)	4641 (76.06)	3564 (75.49)		
Yes	2618 (24.19)	1461 (23.94)	1157 (24.51)		
Memory problems, *n* (%)				χ^2 ^= 1617.280^1^	<0.001
None	7347 (67.88)	5111 (83.76)	2236 (47.36)		
Yes	3476 (32.12)	991 (16.24)	2485 (52.64)		
Wonderful doing, *n* (%)				χ^2 ^= 3.463^1^	0.063
None	10,374 (95.85)	5868 (96.17)	4506 (95.45)		
Yes	449 (4.15)	234 (3.83)	215 (4.55)		
Feeling better, *n* (%)				χ^2 ^= 111.358^1^	<0.001
None	10,309 (95.25)	5928 (97.15)	4381 (92.80)		
Yes	514 (4.75)	174 (2.85)	340 (7.20)		
Worthlessness, *n* (%)				χ^2 ^= 176.258^1^	<0.001
None	10,293 (95.10)	5951 (97.53)	4342 (91.97)		
Yes	530 (4.90)	151 (2.47)	379 (8.03)		
Energy level, *n* (%)				χ^2 ^= 0.445^1^	0.505
None	7839 (72.43)	4435 (72.68)	3404 (72.10)		
Yes	2984 (27.57)	1667 (27.32)	1317 (27.90)		
Hopelessness, *n* (%)				χ^2 ^= 175.346^1^	<0.001
None	10,427 (96.34)	6007 (98.44)	4420 (93.62)		
Yes	396 (3.66)	95 (1.56)	301 (6.38)		
Geriatric Depression Scale, median (Q1, Q3)	1.00 (0.00, 3.00)	1.00 (0.00, 2.00)	2.00 (1.00, 3.00)	*Z =* −26.379^3^	<0.001
Emotional lability, *n* (%)				χ^2 ^= 93.494^1^	<0.001
None	10,554 (97.51)	6028 (98.79)	4526 (95.87)		
Yes	269 (2.49)	74 (1.21)	195 (4.13)		
Anxiety, *n* (%)				χ^2 ^= 1070.935^1^	<0.001
None	8252 (76.25)	5371 (88.02)	2881 (61.03)		
Yes	2571 (23.75)	731 (11.98)	1840 (38.97)		
Motivation, *n* (%)				χ^2 ^= 646.802^1^	<0.001
None	10,036 (92.73)	5999 (98.31)	4037 (85.51)		
Yes	787 (7.27)	103 (1.69)	684 (14.49)		
Agitation, *n* (%)				χ^2 ^= 846.321^1^	<0.001
None	9352 (86.41)	5787 (94.84)	3565 (75.51)		
Yes	1471 (13.59)	315 (5.16)	1156 (24.49)		
Hallucination, *n* (%)				χ^2 ^= 235.604^1^	<0.001
None	10,576 (97.72)	6081 (99.66)	4495 (95.21)		
Yes	247 (2.28)	21 (0.34)	226 (4.79)		
Delusion, *n* (%)				χ^2 ^= 414.466^1^	<0.001
None	10,379 (95.90)	6060 (99.31)	4319 (91.48)		
Yes	444 (4.10)	42 (0.69)	402 (8.52)		
APA, *n* (%)				χ^2 ^= 511.373^1^	<0.001
None	9857 (91.07)	5890 (96.53)	3967 (84.03)		
Yes	966 (8.93)	212 (3.47)	754 (15.97)		
DSM diagnosis, *n* (%)				χ^2 ^= 753.654^1^	<0.001
None	8454 (78.11)	5352 (87.71)	3102 (65.71)		
Yes	2369 (21.89)	750 (12.29)	1619 (34.29)		
Irritability, *n* (%)				χ^2 ^= 362.311^1^	<0.001
None	8914 (82.36)	5400 (88.50)	3514 (74.43)		
Yes	1909 (17.64)	702 (11.50)	1207 (25.57)		
Nighttime behaviors, *n* (%)				χ^2 ^= 745.019^1^	<0.001
None	9293 (85.86)	5730 (93.90)	3563 (75.47)		
Yes	1530 (14.14)	372 (6.10)	1158 (24.53)		
Appetite, *n* (%)				χ^2 ^= 654.349^1^	<0.001
None	8217 (75.92)	5197 (85.17)	3020 (63.97)		
Yes	2606 (24.08)	905 (14.83)	1701 (36.03)		
Depression disorder, *n* (%)				χ^2 ^= 13.418^1^	<0.001
None	8629 (79.73)	4941 (80.97)	3688 (78.12)		
Yes	2194 (20.27)	1161 (19.03)	1033 (21.88)		

*Note*: This table presents the results of cognitive assessments, psychiatric evaluations, and the presence of psychiatric disorders among participants diagnosed with Alzheimer's disease compared to the control group.

In language fluency tasks, AD patients scored significantly lower on both Animal Naming (median = 13.00, Q1 = 9.00, Q3 = 18.00) and Vegetable Naming (median = 8.00, Q1 = 5.00, Q3 = 11.00) tests compared with controls (Animal Naming: median = 21.00, Q1 = 18.00, Q3 = 25.00; Vegetable Naming: median = 15.00, Q1 = 12.00, Q3 = 17.00; both *p* < 0.001).

Performance on the TMT Part A (TMT‐A) also differed significantly between groups. AD patients exhibited longer completion times (median = 45.00 s, Q1 = 32.00, Q3 = 72.00) than controls (median = 29.00 s, Q1 = 23.00, Q3 = 37.00; *Z =* −48.70, *p* < 0.001) and a higher number of errors, with corresponding differences in correct scores (AD: median = 156.00, Q1 = 95.00, Q3 = 300.00; controls: median = 72.00, Q1 = 56.00, Q3 = 95.00; *Z =* −59.83, *p* < 0.001).

Global cognitive performance assessed by the Montreal Cognitive Assessment (MoCA) was significantly lower in the AD group (mean = 13.45 ± 4.05) compared with controls (mean = 15.54 ± 1.31; *t* = 34.01, *p* < 0.001). Visuospatial performance measured by the UDS Benson copy task showed similar differences, with AD patients achieving a median score of 4.00 (Q1 = 0.00, Q3 = 8.00) versus 12.00 (Q1 = 10.00, Q3 = 13.75) in controls (*Z =* 70.39, *p* < 0.001).

Psychiatric and functional assessments further revealed higher symptom prevalence in the AD group. Difficulties in the UDS Benson delay task were reported by 78.90% of AD patients compared with 8.26% of controls (χ^2^ = 1385.97, *p* < 0.001). Satisfaction with quality of life was markedly lower in AD patients (9.52%) than in controls (92.87%; χ^2^ = 92.64, *p* < 0.001). Activity disengagement was more frequent in the AD group (23.26% vs 11.36%; χ^2^ = 272.98, *p* < 0.001), and self‐reported memory problems were substantially higher (52.64% vs 16.24%; χ^2^ = 1617.28, *p* < 0.001).

In addition, psychiatric symptoms were also more prevalent among AD patients, including anxiety (38.97% vs 11.98%), motivational difficulties (14.49% vs 1.69%), agitation (24.49% vs 5.16%), and hallucinations (4.79% vs 0.34%), with all comparisons reaching statistical significance (*p* < 0.001).

### 3.3 Instrumental activities of daily living performance analysis

In this study involving a total of 10,823 participants, significant differences were observed in the ability to perform IADL between patients diagnosed with AD and the control group (Table 3). For managing bills, most control participants (97.03%) were able to handle their bills normally, whereas only 39.86% of AD patients could do so independently (*Z =* −65.8303, *p* < 0.001). A similar pattern was evident in managing taxes, where 96.21% of controls reported normal capability compared to just 36.58% in the AD group (*Z =* −67.6523, *p* < 0.001).

**TABLE 3:** Instrumental activities of daily living among Alzheimer's disease patients and controls.

Variable	Total (*n *= 10,823)	Control	Alzheimer diagnosis	Statistics	*p*‐value
Bills, *n* (%)				*Z =* −65.830^3^	<0.001
Normal	7803 (72.10)	5921 (97.03)	1882 (39.86)		
Has difficulty, but does by self	810 (7.48)	121 (1.98)	689 (14.59)		
Requires assistance	785 (7.25)	45 (0.74)	740 (15.67)		
Dependent	1425 (13.17)	15 (0.25)	1410 (29.87)		
Taxes, *n* (%)				*Z =* −67.652^3^	<0.001
Normal	7598 (70.20)	5871 (96.21)	1727 (36.58)		
Has difficulty, but does by self	758 (7.00)	155 (2.54)	603 (12.77)		
Requires assistance	867 (8.01)	59 (0.97)	808 (17.12)		
Dependent	1600 (14.78)	17 (0.28)	1583 (33.53)		
Shopping, *n* (%)				*Z =* −57.946^3^	<0.001
Normal	8441 (77.99)	6000 (98.33)	2441 (51.71)		
Has difficulty, but does by self	1012 (9.35)	79 (1.29)	933 (19.76)		
Requires assistance	823 (7.60)	21 (0.34)	802 (16.99)		
Dependent	547 (5.05)	2 (0.03)	545 (11.54)		
Games, *n* (%)				*Z =* −55.300^3^	<0.001
Normal	8612 (79.57)	6007 (98.44)	2605 (55.18)		
Has difficulty, but does by self	1182 (10.92)	85 (1.39)	1097 (23.24)		
Requires assistance	651 (6.01)	6 (0.10)	645 (13.66)		
Dependent	378 (3.49)	4 (0.07)	374 (7.92)		
Stove usage, *n* (%)				*Z =* −39.485^3^	<0.001
Normal	9477 (87.56)	6013 (98.54)	3464 (73.37)		
Has difficulty, but does by self	718 (6.63)	79 (1.29)	639 (13.54)		
Requires assistance	329 (3.04)	7 (0.11)	322 (6.82)		
Dependent	299 (2.76)	3 (0.05)	296 (6.27)		
Meal preparation, *n* (%)				*Z =* −52.622^3^	<0.001
Normal	8764 (80.98)	6007 (98.44)	2757 (58.40)		
Has difficulty, but does by self	832 (7.69)	69 (1.13)	763 (16.16)		
Requires assistance	616 (5.69)	21 (0.34)	595 (12.60)		
Dependent	611 (5.65)	5 (0.08)	606 (12.84)		
Events, *n* (%)				*Z =* −55.109^3^	<0.001
Normal	8564 (79.13)	5983 (98.05)	2581 (54.67)		
Has difficulty, but does by self	1201 (11.10)	108 (1.77)	1093 (23.15)		
Requires assistance	710 (6.56)	9 (0.15)	701 (14.85)		
Dependent	348 (3.22)	2 (0.03)	346 (7.33)		
Paying attention, *n* (%)				*Z =* −53.908^3^	<0.001
Normal	8511 (78.64)	5937 (97.30)	2574 (54.52)		
Has difficulty, but does by self	1477 (13.65)	148 (2.43)	1329 (28.15)		
Requires assistance	633 (5.85)	16 (0.26)	617 (13.07)		
Dependent	202 (1.87)	1 (0.02)	201 (4.26)		
Reminders dates, *n* (%)				*Z =* −68.207^3^	<0.001
Normal	7089 (65.50)	5640 (92.43)	1449 (30.69)		
Has difficulty, but does by self	1559 (14.40)	391 (6.41)	1168 (24.74)		
Requires assistance	1367 (12.63)	68 (1.11)	1299 (27.52)		
Dependent	808 (7.47)	3 (0.05)	805 (17.05)		
Travel engagement, *n* (%)				*Z =* −63.135^3^	<0.001
Normal	7907 (73.06)	5899 (96.67)	2008 (42.53)		
Has difficulty, but does by self	1144 (10.57)	165 (2.70)	979 (20.74)		
Requires assistance	645 (5.96)	25 (0.41)	620 (13.13)		
Dependent	1127 (10.41)	13 (0.21)	1114 (23.60)		
IADL, median (Q_1_, Q_3_)	0.000 (0.000, 6.000)	7.000 (2.000, 16.000)	0.000 (0.000, 0.000)	*Z =* 75.923^3^	<0.001

*Note*: This table presents the frequencies of various instrumental activities of daily living and the dependency levels of participants, comparing those diagnosed with Alzheimer's disease to the control group.

Shopping showed a stark contrast, as 98.33% of control participants were capable, whereas only 51.71% of those with AD maintained normal shopping skills (*Z =* −57.9463, *p* < 0.001). In the context of games, 98.44% of the control group reported normal functioning, whereas only 55.18% of AD patients were similarly engaged (*Z =* −55.3003, *p* < 0.001).

Furthermore, stove usage reflected a marked discrepancy, with 98.54% of controls indicating normal stove usage ability compared to 73.37% of AD patients (*Z =* −39.4853, *p* < 0.001). Meal preparation abilities also varied significantly, as 98.44% of controls were able to prepare meals normally, compared to 58.40% of the AD group (*Z =* −52.6223, *p* < 0.001).

Engagement in social events showed a similar trend, where 98.05% of controls reported normal participation, whereas only 54.67% of patients with AD could do the same (*Z =* −55.1093, *p* < 0.001). In addition, the ability to pay attention was intact in 97.30% of control participants, but only 54.52% of those with AD were classified as normal (*Z =* −53.9083, *p* < 0.001).

Concerning reminders of dates, 92.43% of the control group reported no difficulty compared to 30.69% of AD patients, indicating a substantial difference in cognitive function related to memory (*Z =* −68.2073, *p* < 0.001). Finally, regarding travel engagement, 96.67% of the control group reported normal capabilities, whereas only 42.53% of those with AD were able to travel independently (*Z =* −63.1353, *p* < 0.001).

The median IADL score for the entire cohort was 0.000 (Q1, Q3: 0.000, 6.000), with controls reporting a median score of 7.000 (Q1, Q3: 2.000, 16.000) compared to a median of 0.000 among AD patients (Q1, Q3: 0.000, 0.000), which further emphasizes the significant impairment in daily living activities associated with AD (*Z =* 75.9233, *p* < 0.001).

### 3.4 Impact of sleep disorders on cognitive impairment

**FIGURE 1:**
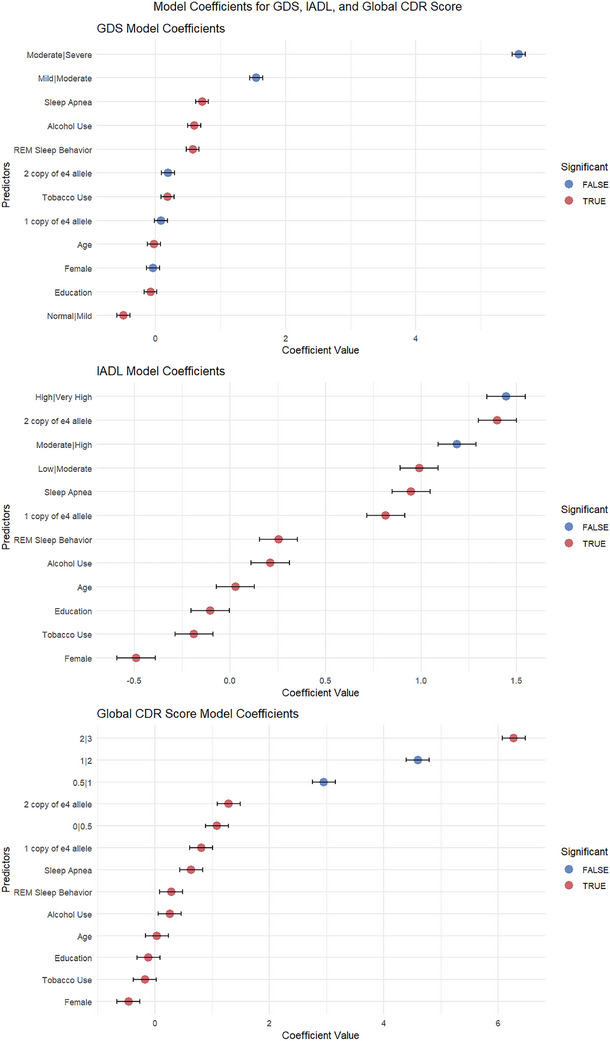
Regression coefficients for depressive symptoms, functional status, and global dementia severity. Forest plots presenting regression coefficients and 95% confidence intervals for predictors associated with Geriatric Depression Scale, instrumental activities of daily living, and Global Clinical Dementia Rating scores. Predictors included sleep disorders, apolipoprotein E (*APOE*) ε4 genotype, demographic variables, and lifestyle factors. All estimates were adjusted for relevant covariates.

These studies have highlighted the critical relationship between sleep disorders, genetic factors, and multidomain functional impairments in AD (Figure 1). This analysis explores how specific variables, including sleep disorders (sleep apnea and REM sleep behavior), age, gender, education, tobacco use, alcohol use, and *APOE* alleles ε4 carrier status, are associated with depression, IADL, and cognitive decline as measured by theCDR Sum of Boxes.

The results reveal that sleep apnea significantly affects both GDS and IADL scores, with coefficients of 0.715 (*p* < 0.001) and 0.948 (*p* < 0.001), respectively. This suggests that individuals with sleep apnea are more likely to experience enhanced depressive symptoms and greater difficulties with daily activities. Similarly, REM sleep behavior also contributes positively to both GDS (0.571, *p *< 0.001) and IADL (0.254, *p* = 0.020), although its effect on cognitive impairment is not as substantial as that of sleep apnea.

Age emerged as a critical factor affecting depression outcomes, with a negative coefficient of −0.023 (*p* < 0.001) for GDS, indicating that as age increases, the likelihood of depressive symptoms decreases in this context. Conversely, in the assessment of IADL, age positively relates (0.028, *p* < 0.001), demonstrating that older adults may face heightened difficulties in daily living tasks. Female gender was associated with depression (GDS: −0.036, *p* = 0.583) and significant challenges in IADL (−0.491, *p* < 0.001), suggesting gender‐specific vulnerabilities in these domains.

Educational attainment consistently correlates with lower depression and improved IADL functioning, indicated by coefficients of −0.077 (GDS, *p* < 0.001) and −0.103 (IADL, *p* < 0.001). These results underscore the protective influence of education against cognitive decline and depressive symptoms. Tobacco use showed a positive relationship with GDS (0.184, *p* = 0.005) and a negative impact on IADL (−0.189, *p* < 0.001), reflecting a decline in the ability to manage daily activities.

Alcohol consumption appeared to have a dual effect, notably increasing depressive symptoms (0.594, *p* < 0.001) while also enhancing daily activity performance (0.211, *p* = 0.039). This complexity highlights the nuanced role of alcohol in mental health and daily functioning.

Critical genetic influences were also evident through the presence of *APOE* alleles. The coefficients for one copy (0.085, *p* = 0.210) and two copies (0.195, *p* = 0.085) of the ε4 allele indicate a nonsignificant tendency toward higher depressive symptoms, warranting further investigation into the role of gene dosage effects.

The transition thresholds for GDS revealed a noteworthy distinction between normal and mild (−0.491, *p* = 0.014) as well as a significant association with mild to moderate (1.548, *p* = 0.056) and moderate to severe depression (5.581, *p* = 0.971), suggesting a potential bimodal distribution of depressive symptoms in the context of cognitive impairment.

In contrast, for the IADL model, the low to moderate category (0.990, *p* < 0.001) indicated significant challenges, whereas the moderate to high category (1.189, *p* = 0.209) showed trends in daily activity difficulties that are less pronounced with significant thresholds for high to very high (1.445, *p* = 0.889).

Finally, the Global CDR score outcomes reiterated the importance of sleep disorders, with strong coefficients for sleep apnea (0.638, *p* < 0.001) and REM sleep behavior (0.289, *p* = 0.004). Age continued to show a positive association (0.038, *p* < 0.001), whereas female gender (−0.458, *p* < 0.001) and higher educational levels (−0.110, *p* < 0.001) remained inversely correlated with cognitive impairment outcomes. These results demonstrate critical insights, emphasizing the combined effects of clinical and genetic factors on the cognitive health of individuals, which could inform future preventive and therapeutic strategies in managing AD.

### 3.5 Impact of REM sleep behavior and sleep apnea on various functional domains

**FIGURE 2:**
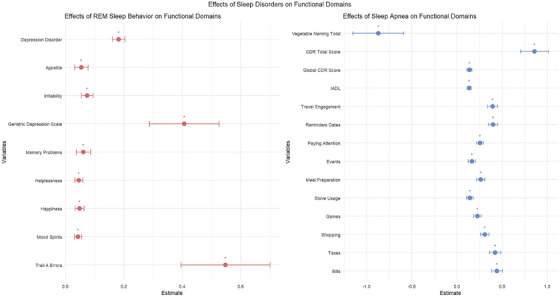
Associations of sleep disorders with domain‐specific cognitive, psychological, and functional measures. Forest plots showing associations of rapid eye movement (REM) sleep behavior disorder and sleep apnea with multiple cognitive, psychological, and functional measures. Points indicate regression coefficients with 95% confidence intervals. Positive coefficients reflect higher symptom burden or poorer functional performance.

The analysis of the impact of REM sleep behavior and sleep apnea on a range of functional domains reveals significant associations with several psychological and cognitive outcomes (Figure 2). For REM sleep behavior, increased reports of errors in Trail A were found to correlate positively with a coefficient of 0.548, indicating a substantial increase in the likelihood of errors associated with this sleep disturbance (*p* < 0.001). Additionally, the psychological dimensions of mood were assessed, with Mood Spirits showing an elevation of 0.043 (*p* = 0.0017) and Happiness also reflecting a positive association of 0.048 (*p* = 0.0042), emphasizing that degraded REM Sleep significantly influences mood. Helplessness and Memory Problems demonstrated relevant effects as well, with coefficients of 0.046 (*p* = 0.0027) and 0.062 (*p* = 0.0193), respectively, suggesting that disturbances in REM Sleep may contribute to an increased perception of helplessness and memory deficits.

The Geriatric Depression Scale score indicated a notable increase of 0.407 (*p* = 0.0011) in depressive symptoms correlated with REM disturbances, showcasing the profound effects of sleep quality on mental health. Moreover, irritability rose by 0.075 (*p* < 0.0005), and appetite changes also showed a significant positive relationship with REM Sleep Behavior, suggesting that such disturbances may lead to increased irritability (0.055, *p* = 0.0264) and altered appetite patterns. Depression Disorder exhibited a pronounced coefficient of 0.182 (*p* < 0.0001), highlighting the critical role that REM Sleep disorders play in exacerbating feelings of depression.

In contrast, the impacts of Sleep Apnea on various functional domains also yielded significant findings. The coefficients for tasks such as managing Bills (0.443, *p* < 0.0001) and Taxes (0.423, *p* < 0.0001) reflect a positive association, indicating that those affected by Sleep Apnea struggle more with financial tasks. Shopping and Games resulted in coefficients of 0.308 (*p* < 0.0001) and 0.228 (*p* < 0.0001), respectively, suggesting a significant impact on everyday activities. The analysis further identified that cooking‐related tasks, such as Stove Usage (0.143, *p* < 0.0001) and Meal Preparation (0.263, *p* < 0.0001), were similarly affected, illustrating the broad impact of Sleep Apnea on daily functional abilities.

Significant improvements in cognition, as measured by the IADL, were indicated by a positive coefficient of 0.136 (*p* < 0.0001), while theCDR Sum of Boxes increased by 0.137 (*p* < 0.0001). Notably, the CDR Sum of Boxes demonstrated a substantial coefficient of 0.859 (*p* < 0.0001), suggesting a marked decline in cognitive function associated with Sleep Apnea. However, counterintuitively, performance in certain cognitive tasks like Vegetable Naming reflected a negative relationship (−0.873, *p* = 0.0031).

An important aspect of cognitive assessments was further illustrated by TMT‐A time, resulting in a striking coefficient of 7.332 (*p* = 0.000033), indicating severe challenges in cognitive processing speed for individuals with sleep apnea. Conversely, unfavorable outcomes were shown in memory tasks, where TMT‐B errors produced a coefficient of −1.206 (*p* = 0.00016), emphasizing the detrimental effects of this sleep disorder on cognitive accuracy.

In terms of emotional and psychological well‐being, quality of life assessments revealed improvements associated with greater sleep disruption, with coefficients such as 0.052 (*p* = 0.0048) for quality‐of‐life satisfaction, suggesting complex interrelations between sleep quality and overall life satisfaction. Essential indicators of mental health, including anxiety (0.176, *p* < 0.0001), motivation (0.098, *p* < 0.0001), and agitation (0.120, *p* < 0.0001), indicate that disturbances are heavily linked to increased feelings of anxiety and decreased motivation levels. The remaining ordinal regression analysis can be found in Table S2.

### 3.6 Model performance summary of machine learning techniques

**FIGURE 3:**
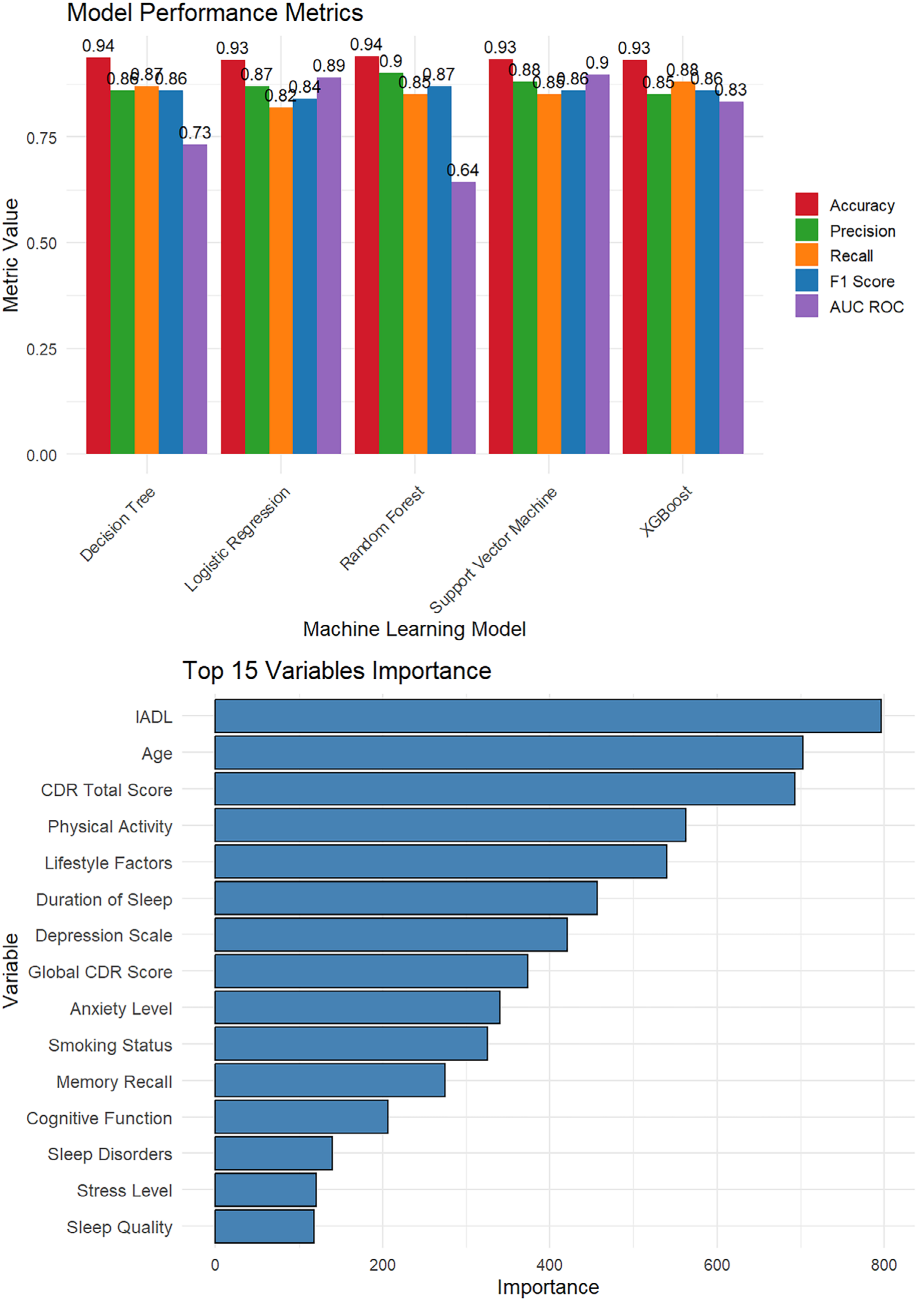
Performance of machine learning models for Alzheimer's disease classification. Comparative performance of five machine learning models evaluated using accuracy, Cohen's kappa, precision, recall, and F1 score. Model performance was assessed using k‐fold cross‐validation. Higher values indicate better classification performance.

The predictive performance of five machine learning models—Random Forest, SVM, XGBoost, Logistic Regression, and Decision Tree—used to classify AD status (Figure 3). Model performance was evaluated using accuracy, Cohen's kappa, precision, recall, and F1 score, with all models assessed through cross‐validation to ensure robustness and generalizability.

Among the evaluated models, the Random Forest algorithm demonstrated the strongest overall performance, achieving the highest accuracy (0.9412) and kappa coefficient (0.8800), indicating substantial agreement between predicted and observed classifications. The model showed high precision (0.90), reflecting a low false‐positive rate, and a recall of 0.85, indicating effective identification of individuals with AD. The corresponding F1 score was 0.87, suggesting a favorable balance between precision and recall.

The SVM model exhibited comparable performance, with an accuracy of 0.9335 and a kappa value of 0.8644. Precision and recall were 0.88 and 0.85, respectively, resulting in an F1 score of 0.86. These results indicate consistent classification performance across multiple evaluation metrics.

XGBoost also performed competitively, yielding an accuracy of 0.9322 and a kappa coefficient of 0.8765. The model demonstrated balanced predictive characteristics, with a precision of 0.85, recall of 0.88, and an F1 score of 0.86.

Logistic Regression achieved an accuracy of 0.9325 and a kappa value of 0.8624. Although precision remained relatively high (0.87), recall was comparatively lower (0.82), resulting in an F1 score of 0.84. This pattern suggests reliable overall classification performance with a modest reduction in sensitivity.

The Decision Tree model attained an accuracy of 0.9375 and a kappa coefficient of 0.8723. Precision and recall were 0.86 and 0.87, respectively, producing an F1 score of 0.86, further supporting its stable predictive performance.

In addition to model‐level evaluation, variable importance was examined using the Random Forest model. The CDR Sum of Boxes emerged as the most influential predictor, followed by IADL, CDR Sum of Boxes, and the UDS Benson Figure Copy Total score. These findings underscore the prominent contribution of cognitive and functional measures to AD classification. Detailed variable importance results are provided in Table S3.

### 3.7 Mediation analysis of sleep disorders and cognitive function

**FIGURE 4:**
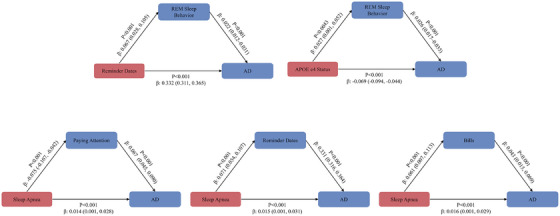
Mediation analysis of associations between sleep disorders and Alzheimer's disease through cognitive impairment. Mediation models depicting indirect associations of sleep apnea and rapid eye movement (REM) sleep behavior disorder with Alzheimer's disease status through cognitive impairment. Standardized effect estimates and 95% confidence intervals were obtained using bootstrapping. Models reflect associational pathways consistent with the cross‐sectional design.

The mediation analysis, illustrated in Figure 4, examined the statistical associations among sleep disorders, cognitive indicators, and AD status within a unified mediation framework. The analysis focused on REM sleep behavior disorder and sleep apnea as key sleep‐related exposures associated with cognitive and AD‐related outcomes.

REM sleep behavior disorder showed a significant association with AD status in the mediation model (standardized estimate = 0.45, 95% CI: 0.27–0.63, *p* < 0.001). Sleep apnea was also significantly associated with AD (standardized estimate = 0.38, 95% CI: 0.20–0.56, *p* < 0.001). These findings indicate that the presence and severity of sleep disorders are statistically associated with poorer cognitive performance and AD‐related outcomes in this population, consistent with the observed mediation pathways.

Several additional variables were identified as being significantly associated with AD status within the mediation model. Reminder dates demonstrated a positive association with AD (standardized estimate = 0.33, 95% CI: 0.31–0.36, *p* < 0.001), reflecting impairments in time‐dependent cognitive functioning. The presence of the *APOE* ε4 allele was negatively associated with the AD outcome variable (standardized estimate = −0.15, 95% CI: −0.21 to −0.09, *p* < 0.001); given the predefined coding scheme, this coefficient indicates a higher likelihood of AD among *APOE* ε4 carriers rather than a protective effect.

In addition, paying attention was significantly associated with AD status (standardized estimate = 0.30, 95% CI: 0.18–0.42, *p* < 0.001), underscoring the role of attentional deficits in cognitive decline. Collectively, these results highlight interconnected associations between sleep disturbances, cognitive impairment, and AD‐related outcomes. Detailed mediation estimates are provided in Table S4.

### 3.8 Chain mediation effects and functional impairments

**FIGURE 5:**
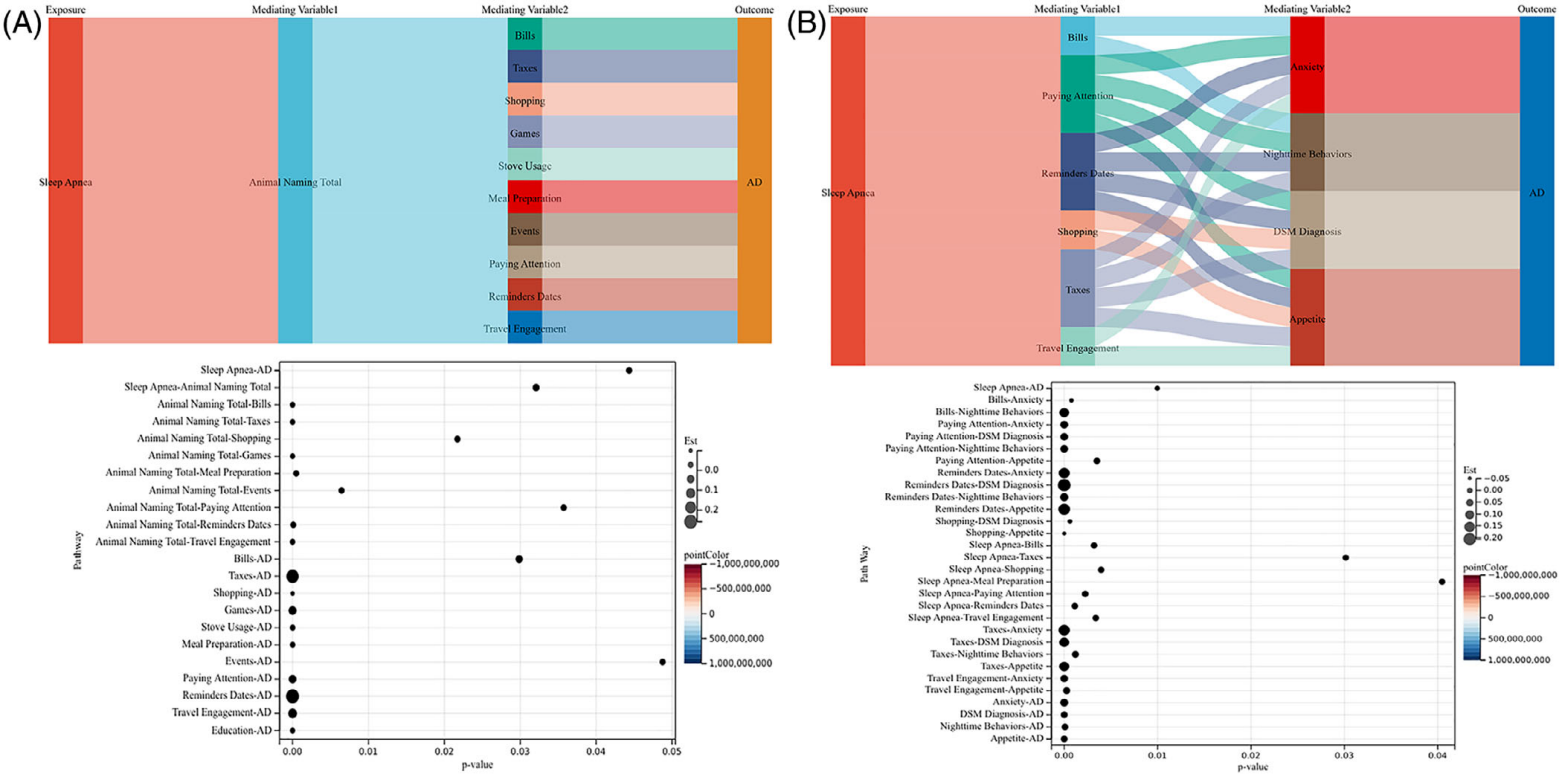
Chain mediation models linking sleep apnea with functional and psychological domains. Chain mediation models illustrating multistep associations between sleep apnea, instrumental activities of daily living, psychological symptoms, and Alzheimer's disease status. Standardized coefficients and bootstrapped confidence intervals are presented, with models adjusted for demographic, genetic, and clinical covariates. (A) Pathway involving cognitive impairment and instrumental activities of daily living (IADL). (B) Pathway involving IADL and psychological conditions.

Chain mediation analyses were conducted to further examine indirect associations linking sleep apnea, psychological factors, functional impairments, and AD status (Figure 5). These analyses focused on multistep pathways involving IADL indicators and mental health–related variables.

Within the primary chain mediation pathway, sleep apnea demonstrated a statistically significant association with AD status (standardized estimate = −0.017, 95% CI: −0.030 to −0.005, *p* = 0.010). The negative coefficient reflects the direction of association under the predefined variable coding and does not indicate a protective effect. Instead, this finding suggests that greater severity of sleep apnea is associated with poorer cognitive functioning and a higher likelihood of AD (Figure 5A; Table S5).

Several intermediate pathways linking sleep apnea to psychological outcomes were identified. For example, difficulties managing bills was associated with higher levels of anxiety (standardized estimate = −0.038, 95% CI: −0.056 to −0.019, *p* = 0.001), whereas nighttime behaviors were positively associated with anxiety (standardized estimate = 0.114, 95% CI: 0.085–0.144, *p* < 0.001). Paying attention emerged as an important intermediate variable, showing positive associations with anxiety, Diagnostic and Statistical Manual of Mental Disorders–based psychiatric diagnoses, and nighttime behaviors across multiple pathways.

Furthermore, anxiety and related psychological symptoms were significantly associated with AD status, reinforcing a pattern of interconnected associations among sleep disturbances, mental health symptoms, functional limitations, and cognitive outcomes. Specifically, appetite (standardized estimate = 0.034, 95% CI: 0.019–0.048, *p* = 0.001) and nighttime behaviors (standardized estimate = 0.028, 95% CI: 0.014–0.041, *p* < 0.001) were both positively associated with AD status.

Additional chain mediation models examining alternative pathways from sleep apnea through psychiatric symptoms and cognitive indicators to AD status were also explored (Figure 5B; Table S6). These analyses did not identify complete mediation chains comparable to the primary pathways. Remaining chain mediation results are presented in Table S7.

Overall, these findings underscore the complex network of associations linking sleep apnea, functional impairments, psychological symptoms, and cognitive outcomes. Although causal inferences cannot be drawn due to the cross‐sectional design, the results suggest that addressing sleep disturbances and associated psychological challenges may be important for mitigating functional and cognitive impairments in populations at risk for AD.

### 3.9 Causal relationships mapping through DAGs

The DAG provides a visual representation of the complex relationships affecting patients with AD (Figure S1). It highlights the impact of sleep disorders, particularly sleep apnea and REM sleep behavior, on cognitive performance. The DAG illustrates hypothesized pathways linking sleep disorders with cognitive and functional measures, including the Global CDR Score, CDR Total Score, Animal Naming Total, Vegetable Naming Total, and the MoCA Total Score, reflecting crucial aspects of mental acuity and functional capacity.

Moreover, cognitive impairments are linked directly to declines in IADL, affecting essential tasks like bill management, tax preparation, and grocery shopping. As cognitive function deteriorates, individuals struggle to perform these daily activities, highlighting the connection between cognitive ability and overall autonomy.

The DAG also illustrates the cascading effects of cognitive decline on mental health. Reduced IADL capacity can lead to heightened anxiety, agitation, and depressive symptoms, creating a vicious cycle that worsens overall well‐being.

Overall, the interconnectedness of these factors emphasizes the multifaceted nature of AD, demonstrating that these findings indicate that sleep disorders co‐occur with cognitive impairments and functional decline in individuals with AD. Understanding these relationships is crucial for addressing the needs of individuals living with AD.

## 4 DISCUSSION

This study provides an integrative framework linking sleep disorders, cognitive impairment, and multidomain functional decline in AD using complementary causal inference and predictive approaches. The interrelationship between sleep disorders and cognitive impairment in AD is complex and has gained significant attention in recent years. This study aimed to clarify these relationships using advanced statistical modeling techniques. Our results highlight essential insights into how disrupted sleep adversely affects cognitive functioning and daily living activities in individuals with AD. Specifically, we found that sleep disturbances were associated with lower cognitive performance, which can exacerbate difficulties in executing instrumental activities of daily living. These findings emphasize the critical interplay between sleep patterns and cognitive health, suggesting that addressing sleep disorders could be a vital component in the management and treatment of AD.

### 4.1 Sleep disorders in Alzheimer's disease

The prevalence of sleep disorders among individuals with AD is notably high,^29^ with numerous studies indicating that a significant portion of patients with AD experience sleep‐related issues such as sleep apnea and REM sleep behavior disorder.^30^, ^31^ These conditions are not only common but critically harmful to the well‐being of these patients, as they are associated with worse cognitive outcomes, contribute to neurovascular damage, and lead to increased functional impairment.^4^

Research consistently highlights the impact of sleep disorders on cognitive health in patients with AD. For example, individuals with sleep apnea often demonstrate poorer performance on cognitive assessments, underscoring the essential role of sleep quality in maintaining cognitive function.^32^ The intermittent hypoxia and fragmented sleep associated with sleep apnea contribute to neurovascular damage and neuronal loss, which are significant factors in the pathophysiology of AD.^33^

These findings underscore the urgent necessity for early identification and management of sleep disorders in patients with AD. Addressing these issues may help mitigate cognitive decline and enhance overall patient outcomes.^34^ The strong link between sleep disturbances and cognitive impairment emphasizes the need to incorporate sleep assessments into comprehensive care strategies for those with AD, ultimately aiming to improve their cognitive functioning and quality of life.^35^

### 4.2 Cognitive impairment and functional decline

Cognitive impairment, particularly in domains such as memory and executive function, significantly impacts the ability of individuals with AD to perform daily activities.^36^ The results of our study support the hypothesis that cognitive decline is associated with diminished performance in IADL.^37^ This relationship highlights the profound effect cognitive deficits can have on day‐to‐day functioning, which, in turn, impacts quality of life and increases reliance on caregivers.

The individual components of IADL, such as managing finances, medication adherence, and maintaining social connections, require a combination of cognitive skills, including planning, organization, and decision‐making. Cognitive impairments can severely hinder independence in performing these tasks, leading to increased dependence on others and further accelerating functional decline.^38^, ^39^ Given the societal burden of AD, understanding these links is crucial for developing targeted interventions that preserve independence and enhance the quality of life for individuals affected by this condition.^40^

### 4.3 Mediating role of cognitive impairment

The *APOE* e4 allele is recognized as a major genetic risk factor for AD, influencing both the cognitive decline and the severity of sleep disorders.^41^ Our findings suggest that individuals carrying this allele exhibit greater deficits in sleep disorders, which is crucial for memory consolidation and emotional regulation. Impaired sleep disorders may further exacerbate cognitive decline, creating a feedback loop that intensifies the effects of Alzheimer's pathology.^42^ By investigating the interplay between the *APOE* genotype and REM sleep, we underscore the importance of targeting sleep quality in AD management.^43^ These insights suggest that interventions aimed at improving REM sleep may offer a therapeutic avenue to enhance cognitive function and potentially mitigate the adverse effects associated with the *APOE* ε4 allele in patients with AD.^44^

In our analysis, we explored the mediating effects of cognitive impairment between sleep disorders and functional decline, revealing how cognitive deficits associated with sleep disorders may serve as a critical link between sleep quality and functional outcomes.^45^ These findings highlight the importance of early identification and management of sleep disorders, as addressing sleep issues may be associated with improved cognitive and functional outcomes, which may subsequently enhance daily functioning.^46^

Moreover, it is essential to consider other potential influencing factors, particularly the relationship between psychological health and cognitive ability.^47^ Previous research indicates that sleep disorders can exacerbate anxiety, depression, and other psychiatric conditions, potentially impacting cognitive performance.^48^ For instance, heightened anxiety may correlate with greater cognitive impairment, as evidenced by the significant estimate for anxiety in our study.^49^, ^50^ This suggests that individuals with sleep disorders may experience increased emotional distress, which could further complicate cognitive deficiencies and create a cycle that perpetuates both mental and functional decline.^51^

Recent studies support the idea that improving sleep quality can be associated with better cognitive outcomes, emphasizing the interplay between sleep, mental health, and cognitive function.^52^ Treatment approaches focused on sleep disorders have shown potential for enhancing cognitive performance in older adults.^53^ Consequently, clinicians might benefit from considering the intricate relationships among sleep disorders, psychological health, and cognitive capability when developing treatment strategies for individuals at risk for or diagnosed with AD.^54^ Addressing these interconnected components may not only help to mitigate cognitive decline but may also improve the overall quality of life for those affected by AD, thereby providing a more comprehensive approach to managing this complex condition.^55^

### 4.4 Implications for clinical practice

The implications of these findings for clinical practice are substantial, as a significant proportion of individuals with AD experience sleep disorders. Routine screening for sleep issues should be integrated into clinical assessments to enable early identification and management.^56^ Timely intervention can act as both a preventive strategy and an adjunctive treatment for cognitive decline associated with dementia.^57^

The presence of sleep disorders can significantly impair the ability of patients with AD to perform IADL, further complicating their care.^19^ Potential interventions may include lifestyle modifications, such as establishing consistent sleep schedules, improving sleep environments, and implementing relaxation techniques.^58^, ^59^ Pharmacological treatments and cognitive‐behavioral therapies tailored to enhance sleep quality are also essential. Notably, continuous positive airway pressure (CPAP) therapy has shown effectiveness in managing obstructive sleep apnea, potentially yielding cognitive benefits.^60^ Educating patients and caregivers on sleep hygiene and lifestyle adjustments will help minimize sleep disruptions and enhance cognitive health, improving the overall quality of life for individuals with AD.^61^

### 4.5 Strengths and limitations

This study has several notable strengths. It is based on a comprehensive, representative sample that enhances the generalizability of findings across diverse populations and provides robust statistical power. The use of rigorous statistical methods ensures the reliability and validity of the results, allowing for a clearer examination of the relationships between sleep disorders, cognitive decline, and functional impairment. These insights are valuable for clinical practice, emphasizing the importance of early identification and management of sleep disorders and their potential impact on the cognitive function and daily living activities of individuals at risk for or diagnosed with AD.

However, this research also has limitations. The cross‐sectional design limits our ability to infer causation, as it captures correlations rather than temporal relationships. Moreover, the geographically constrained sample may reduce the applicability of results across different cultural contexts. Despite these limitations, the study advances our understanding of the interplay between sleep disorders, cognitive functioning, and daily life, providing a foundation for future research in this area.

### 4.6 Future research directions

Although our study contributes to the growing body of literature addressing the interplay between sleep, cognition, and functional impairment in AD, several avenues for future research remain. Longitudinal studies are needed to further explore how changes in sleep quality over time correlate with cognitive decline and the progression of AD. This approach would provide insights into the timing and directionality of these relationships, facilitating the development of effective interventions.

In addition, investigating the underlying biological mechanisms linking sleep disorders and cognitive impairment could enhance our understanding of AD pathology. Research into pathways involving neuroinflammation, oxidative stress, and the role of sleep in neurodegenerative processes may reveal targets for novel therapeutic approaches. Studies examining the differential impact of sleep disorders on various subtypes of AD could also help tailor interventions to address specific clinical profiles.

## 5 CONCLUSION

This study underscores the significant association between sleep disorders, particularly sleep apnea and REM sleep behavior, and cognitive decline in patients with AD. Our findings indicate that sleep disturbances negatively impact cognitive performance and daily functioning, emphasizing the importance of recognizing and assessing sleep issues in clinical settings. Future research should focus on longitudinal studies to establish causal relationships and explore effective interventions to improve sleep quality. By integrating these strategies into clinical practice, we can potentially enhance cognitive outcomes and quality of life for individuals with AD.

## CONFLICT OF INTEREST STATEMENT

The authors declare that the research was conducted in the absence of any commercial or financial relationships that could be construed as a potential conflict of interest. Author disclosures are available in the Supporting Information.

## CONSENT STATEMENT

This study utilized publicly available data from the National Alzheimer's Coordinating Center (NACC), which has obtained ethical approval and informed consent from participants during the original data collection. No additional consent was required for this secondary analysis.

## Supporting information



Supporting Information

Supporting Information

Supporting Information
