# Supplementary material for: Waking up to the truth: Associations between sleep disorders and multidomain functional outcomes in Alzheimer's disease
Source: Alzheimers Dement. 2026 Feb 24;22(2):e71200. doi: 10.1002/alz.71200 (PMC12930103; doi:10.1002/alz.71200)
Supplement: Supplementary file 1 — Supporting Information [file ALZ-22-e71200-s003.pdf]

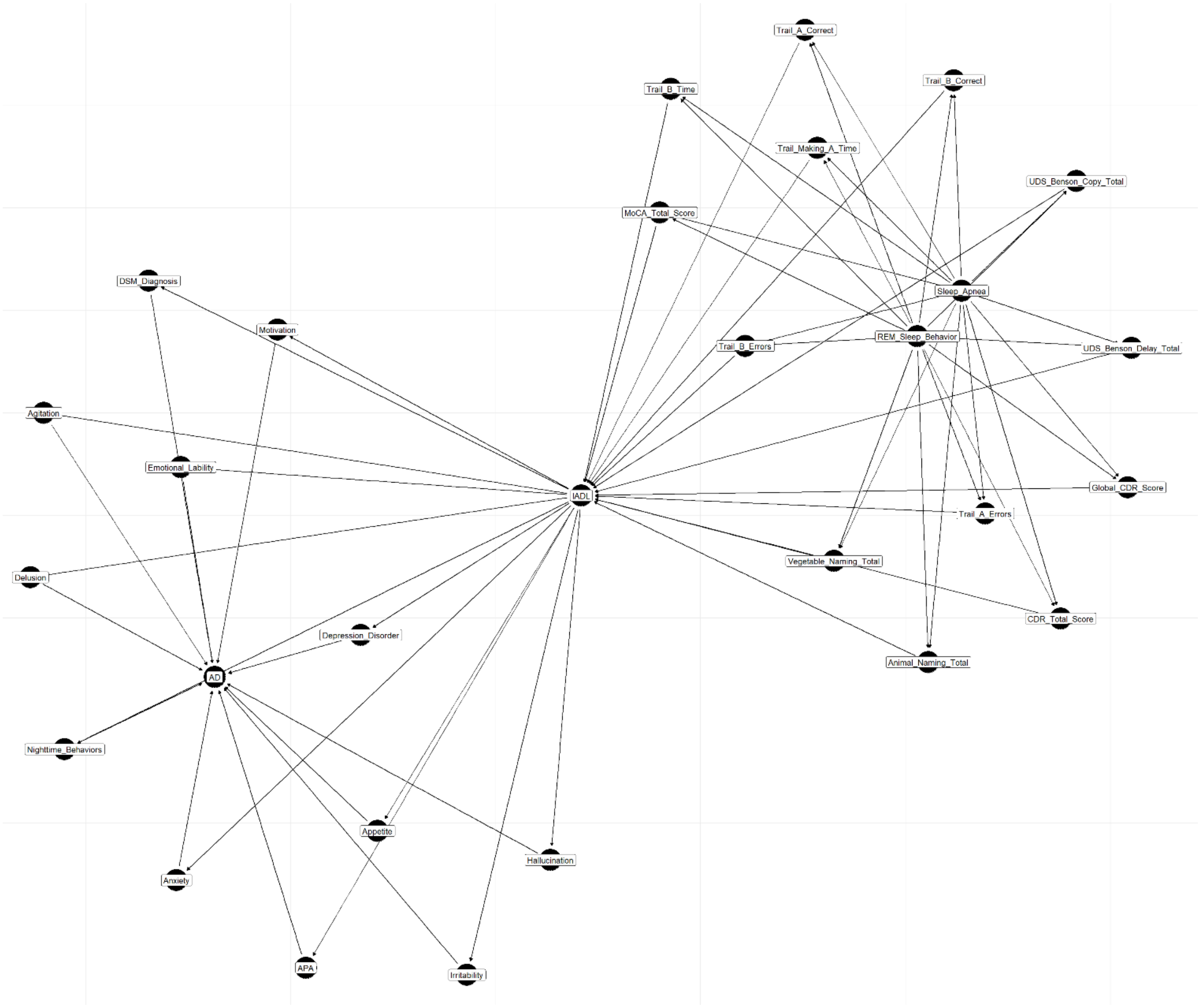

Figure S1: Directed Acyclic Graph Analysis of Causal Relationships among Sleep Disorders and Multidomain Functionality in Alzheimer' s disease
